# Assessment of Neuromuscular Blockade Depth Using Train-of-Four (TOF) Monitoring Following Rocuronium Extravasation

**DOI:** 10.7759/cureus.91997

**Published:** 2025-09-10

**Authors:** Keitaro Yoshioka, Tsuwa Iwamoto, Hirokazu Kawase, Shinichi Ishikawa, Yoshimasa Takeda

**Affiliations:** 1 Department of Anesthesiology, Toho University Omori Medical Center, Tokyo, JPN

**Keywords:** first twitch height, recurarization, rocuronium bromide, rocuronium extravasation, sugammadex, train-of-four, train-of-four (ratio)

## Abstract

This report describes a case of accidental rocuronium extravasation in a patient with end-stage renal disease. We were unable to assess the depth of neuromuscular blockade due to fluctuating train-of-four (TOF) counts ranging from 0 to 4 between 37 and 90 minutes post-first rocuronium administration. However, the first twitch height in TOF stimulation (T1) was persistently low during this period and appeared to reflect a deep level of neuromuscular blockade. Thereafter, T1 increased steadily in response to rising TOF count and TOF ratio. In our case, T1 was low on average during TOF count fluctuation, at 2 ± 2% (mean ± standard deviation). Between 91 and 105 minutes post-first rocuronium administration, TOF counts of 4 were observed more frequently, and the mean T1 increased to 7 ± 4%. Beginning at 106 minutes post-first rocuronium administration, the TOF count remained constant at 4, and the mean T1 increased to 9 ± 3%. Beginning at 135 minutes, the TOF ratio appeared to be constant, and the mean T1 increased to 18 ± 5% until 162 minutes. At 185 minutes post-first rocuronium administration, we administered sugammadex 200 mg (3.17 mg/kg) after the patient’s breathing had recovered spontaneously (end-tidal CO₂ was 42 mmHg, tidal volume exceeded 6 mL/kg, and respiratory rate was 12 breaths/min). The patient experienced no adverse events such as postoperative recurarization. In our case, although the TOF count was unstable, T1 appeared to reliably reflect changes in the depth of neuromuscular blockade.

## Introduction

Rocuronium extravasation causes prolonged neuromuscular blockade. To date, six cases have been reported in the literature [1-6]. While five cases followed a typical course of train-of-four (TOF) count recovery [1-5], one case exhibited unusual TOF count fluctuations [6]. In that case, the TOF count fluctuated between 0 and 3 for 105 minutes until sugammadex was administered. Although the TOF count increased to 4 following the administration of sugammadex, recurarization was observed. Assessing the depth of neuromuscular blockade is crucial, particularly in cases of rocuronium extravasation. In our case, we experienced TOF count fluctuation. Although TOF count fluctuation led to inconsistent assessments of neuromuscular blockade, the first twitch height in TOF stimulation (T1) was persistently low and within a stable range throughout this period. Previous studies have shown that T1 correlates quantitatively with both TOF count and TOF ratio, making it a simple and consistent indicator of neuromuscular blockade depth [7,8]. This consistency suggests that T1 may serve as a reliable indicator of neuromuscular blockade under such conditions. Herein, we report the time course of T1, TOF count, and TOF ratio in this rocuronium extravasation case.

## Case presentation

A 50-year-old man (body weight: 63 kg) with a 30-year history of hemodialysis was scheduled to undergo occipitocervical fixation. Anesthesia was induced via an intravenous line placed in the left forearm with propofol (150 mg), fentanyl (100 μg), and rocuronium 0.79 mg/kg (50 mg). However, extravasation was identified when limited mouth opening was observed even after the administration of rocuronium, prior to intubation.

An additional 50 mg of rocuronium was administered through a newly secured intravenous line. No further rocuronium was administered thereafter, and anesthesia was maintained with desflurane and remifentanil.

At 37 minutes post-first rocuronium administration, neuromuscular blockade monitoring was initiated, considering the prolonged effect of subcutaneous rocuronium. Neuromuscular monitoring was performed at the adductor pollicis muscle, with stimulating electrodes placed along the course of the ulnar nerve (Neuromuscular Module™ AF-101; Nihon Kohden, Tokyo, Japan).

**Figure 1:**
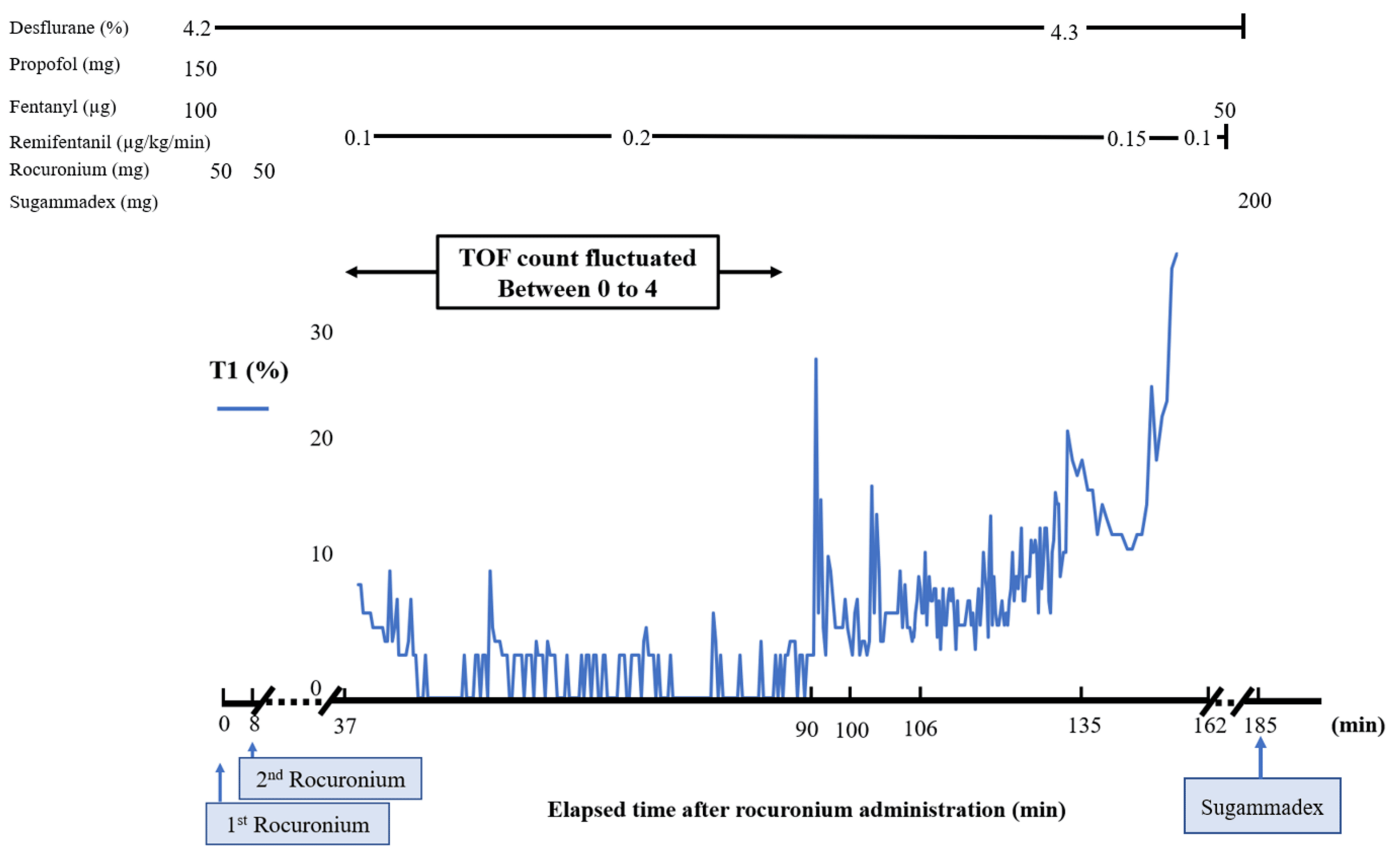
Time course of the first twitch height in TOF stimulation (T1) and anesthetic dosage The value in parentheses represents means ± standard deviations. This figure illustrates the time course of the first twitch height in TOF stimulation (T1) in response to changes in the depth of neuromuscular blockade. From 37 to 90 minutes, corresponding to the period of TOF count fluctuation, T1 was low on average (2 ± 2%). From 91 to 105 minutes, the mean T1 increased to 7 ± 4%. Beginning at 106 minutes, the mean T1 gradually increased to 9 ± 3% by 135 minutes. Beginning at 135 minutes, the TOF ratio appeared constant, and the mean T1 increased to 18 ± 5% until 162 minutes. TOF: train-of-four

**Figure 2:**
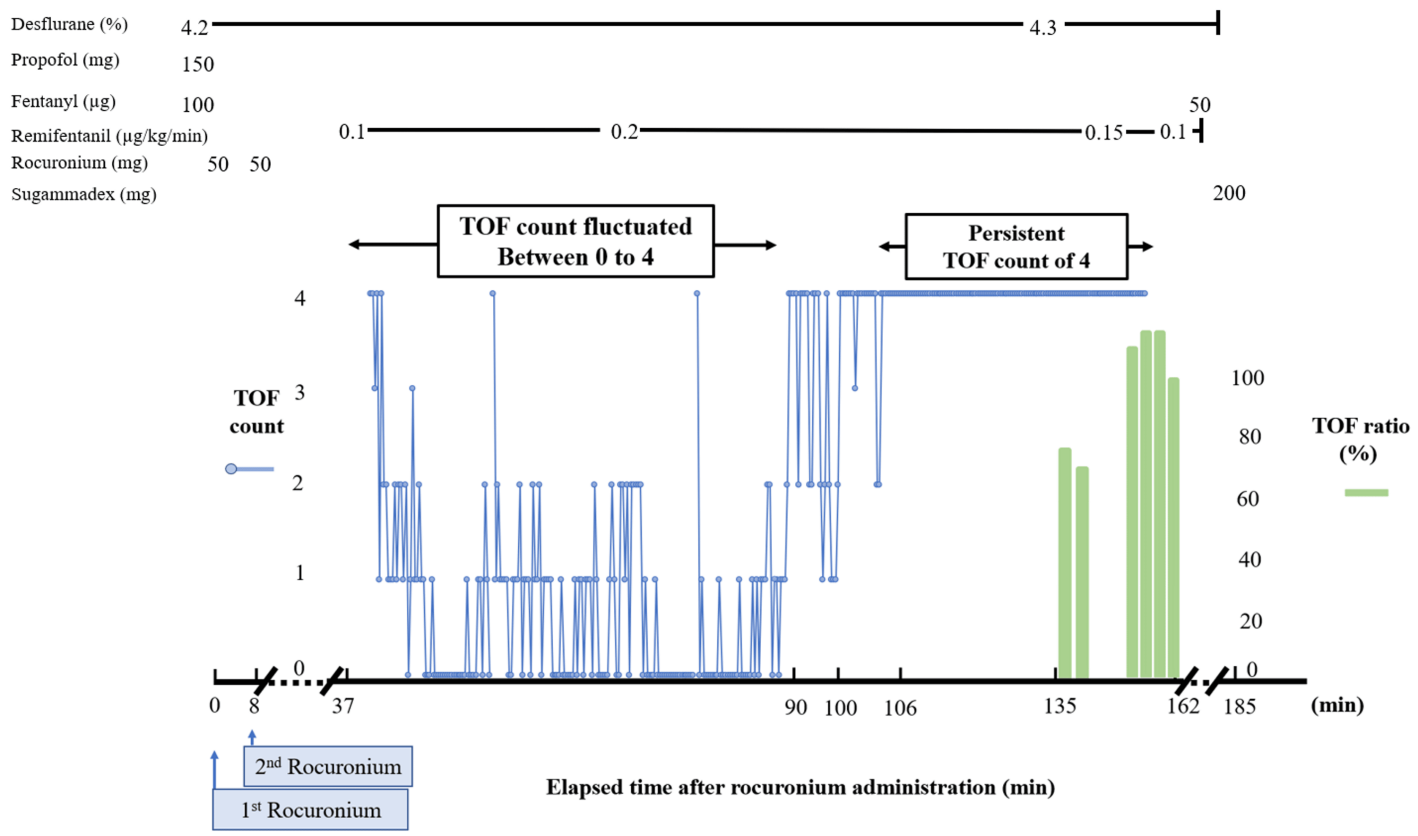
Time course of TOF count, TOF ratio, and anesthetic dosage TOF count fluctuated between 0 and 4 irregularly until 90 minutes. From 91 to 105 minutes, a TOF count of 4 was observed more frequently. From 106 min, the TOF count remained consistently at 4. The TOF ratio was first displayed at 73% at 135 minutes. Eventually, the TOF ratio displayed 94% at 162 minutes. TOF: train-of-four

The TOF monitor used in this case was an acceleromyography-based device that measures the acceleration of thumb movement in response to ulnar nerve stimulation. The time course of T1 is shown in Figure 1, and the time courses of TOF count and TOF ratio are shown in detail in Figure 2. Intraoperative temperature was maintained between 36.1°C and 36.6°C. No intraoperative neurophysiological monitoring, such as motor evoked potentials or somatosensory evoked potentials, was performed.

At 162 minutes, the final TOF measurement revealed a T1 of 33% and a TOF ratio of 94%. At 185 minutes, spontaneous breathing recovery (tidal volume >6 mL/kg, respiratory rate 12 breaths/min) was confirmed, and sugammadex 200 mg (3.17 mg/kg) was administered, followed by extubation. After approximately one hour of observation, the patient was transferred to the ward from the operating room without complications. Postoperatively, no signs of recurarization were noted, and hemodialysis was resumed on postoperative day 1. Written informed consent for publication of this case and the accompanying images was obtained from the patient.

**Table 1:** Summary of previously reported cases of rocuronium extravasation TOF: train-of-four

References	Rocuronium (mg)	Weight (kg)	Complications	TOF count before sugammadex administration	Sugammadex (mg/kg)	Recurarization
							1	70 mg	67 kg	Hemodialysis	2 (stable)	4.5 mg/kg	No
2	100 mg	86 kg	Hemodialysis	1 (stable)	4 mg/kg	No							
							3 (case 1 only)	70 mg	65 kg	Hepatic trauma	4 (stable)	2.31 mg/kg	No
4	50 mg	82 kg	None	4 (unknown)	2.0 mg/kg	No							
							5	100 mg	120 kg	Stroke	2 (unknown)	2.0 mg/kg	No
6	225 mg	73.5 kg	None	1 (fluctuating)	2.72 mg/kg	Yes							
							This case	100 mg	63.5 kg	Hemodialysis	4 (stable)	3.17 mg/kg	No

## Discussion

In our case, a patient with renal failure who had been receiving dialysis treatment experienced an accidental administration of rocuronium both subcutaneously and intravenously (50 mg each) during anesthesia induction. Notably, considering the risk of high rocuronium concentration being associated with no renal excretion and supply from a subcutaneous depot, monitoring the depth of neuromuscular blockade is imperative for determining the timing of sugammadex administration and extubation.

First twitch height in TOF stimulation (T1)

In our case, T1 seemed to reflect the change of neuromuscular blockade in a scalable manner. It has been reported that TOF counts of 1, 2, 3, and 4 correspond to T1 of 8 ± 4%, 20 ± 6%, 33 ± 9%, and 44 ± 10%, respectively [7]. Additionally, the TOF ratios of 70% and 90% correspond to T1 of 69 ± 8% and 86 ± 5%, respectively [8]. These findings indicate that T1 can serve as a simple and consistent indicator that scalably reflects the change of neuromuscular blockade from profound (TOF count 1) to shallow (TOF ratio 90%) levels. In our case, the scalable changes in T1 are illustrated in Figure 1. T1 may serve as an effective indicator for assessing neuromuscular blockade throughout the entire period in patients with rocuronium extravasation.

TOF count

In our case, we experienced TOF count fluctuation between 0 and 4 between 37 and 90 minutes post-first rocuronium administration. Although the exact mechanism underlying these fluctuations remains unknown, it may involve the TOF counting algorithm. TOF count is defined as the number of successive times that the twitch height rises to ≥3%. For example, if a set of T1, T2, T3, and T4 heights is 5%, 2%, 3%, and 3%, respectively, the TOF count is 1, because the T2 (i.e., the second twitch height during TOF stimulation) did not exceed the threshold. In our case, at 38 minutes post-first rocuronium administration, the TOF count was 4, and the heights of T1, T2, T3, and T4 were 6%, 4%, 3%, and 3%, respectively. Since all of the twitch heights remained near the counting threshold, even small changes in twitch heights could significantly affect the number of TOF counts.

TOF ratio

In our TOF monitor algorithm, the TOF ratio is displayed when the T1 is ≥20%. In the present case, since T1 consistently exceeded 20% from 135 minutes post-first rocuronium administration, the TOF ratio appeared in the later phase of neuromuscular recovery. Consequently, the TOF ratio appeared only during the later phase of neuromuscular recovery and was therefore useful only for assessing neuromuscular blockade during a limited phase of recovery.

Cause of TOF count fluctuation

In our case, the prolonged fluctuation period (37-90 minutes post-first rocuronium administration) may be explained by a temporary equilibrium between rocuronium supply and elimination, resulting from sustained rocuronium release from the subcutaneous depot and the absence of renal excretion. Since all twitch heights remained near the counting threshold during this period, even small changes in twitch heights could significantly affect the TOF count. These factors likely contributed to the observed instability in the TOF count.

Limitations

In our case, we did not calibrate the TOF monitor. The purpose of calibration is to detect the maximum thumb acceleration in each patient [9]. This number is used as the control value. T1 is calculated as the ratio of the actual value to the control value. Without calibration, the control value is set to the maximum acceleration that the monitor can detect. Since this theoretical control value is higher than the actual control value, omitting calibration tends to result in lower T1 values than they truly are. However, in this case, T1 showed a gradual increase consistent with the progression of neuromuscular recovery, as reflected in TOF count and TOF ratio monitoring. Although the absolute T1 value may not have been accurate due to the lack of calibration, T1 reflected changes in neuromuscular blockade in a scalable manner.

## Conclusions

We were able to assess the change of neuromuscular blockade of a dialysis patient with rocuronium extravasation. Although the fluctuating TOF count led to inconsistent assessments of neuromuscular blockade, T1 was low during this period and then increased steadily in a scalable manner thereafter. Our experience with this case suggests that T1 may represent a reliable and scalable indicator of neuromuscular blockade changes in situations involving rocuronium extravasation.
